# A retrospective study of the clinical efficacy of arthroscopic double-row and single-row suture-bridge techniques for rotator cuff tears

**DOI:** 10.1007/s00423-026-04212-1

**Published:** 2026-08-19

**Authors:** Bingqiang Shi, Bingling Shi, Yong Xu

**Affiliations:** 1 YunnanPùer Medical School, No. 29, Biancheng East Road, Pu’er City, Yunnan Province 665000 China; 2 Department of Medical Imaging, Hunan University of Medicine General Hospital, 144 Jinxi South Road, Hecheng District, Huaihua City, 418000 PR China; 3 Department of Bone Diseases and Sports Medicine, Hunan University of Medicine General Hospital, 144 Jinxi South Road, Hecheng District, Huaihua City, 418000 PR China

**Keywords:** Rotator cuff tear, Arthroscopic surgery, Single-row repair, Double-row repair

## Abstract

**Objective:**

The comparative efficacy of single-row (SR) and double-row (DR) suture-bridge techniques in arthroscopic rotator cuff repair remains controversial, particularly with regard to optimal patient selection. This study aimed to compare the early clinical outcomes of the two techniques and, importantly, to perform subgroup analyses according to tear size to identify the patient populations most likely to benefit from the DR technique.

**Methods:**

A total of 144 patients were retrospectively assigned to the DR group (n = 73) or SR group (n = 71) according to the surgical technique used. Perioperative parameters, visual analogue scale (VAS) scores, Constant-Murley scores (CMS), American Shoulder and Elbow Surgeons (ASES) scores, University of California at Los Angeles (UCLA) shoulder scores, and range of motion (ROM) were compared between the two groups before surgery and at the final follow-up. Magnetic resonance imaging (MRI) was performed 12 months postoperatively to assess the rotator cuff retear rate. Subgroup analyses were conducted after stratification into small-to-medium (SM) and large-to-massive (LM) tear groups.

**Results:**

Operative time and intraoperative blood loss were greater in the DR group than in the SR group, with statistically significant differences between the groups (P = 0.007). Postoperatively, functional scores and VAS scores improved significantly from baseline in both groups (P < 0.05). Compared with the SR group, the DR group demonstrated significantly greater improvements in VAS, CMS, UCLA, and ASES scores (all P < 0.001). Assessment of shoulder ROM and imaging outcomes also showed significantly greater improvements in the DR group than in the SR group (P < 0.05). Subgroup analysis revealed that the functional benefits of the DR technique were primarily attributable to patients with LM tears, in whom the improvements exceeded the minimal clinically important difference (MCID). In contrast, for patients with SM tears, the clinical relevance of the observed between-group differences was less definitive.

**Conclusion:**

Arthroscopic double-row suture-bridge repair is associated with superior early functional recovery and a lower retear rate than the single-row technique. However, these benefits appear to be driven primarily by outcomes in patients with large-to-massive tears. For small-to-medium tears, the clinically meaningful advantage of the DR technique is less evident, suggesting that tear size may be an important determinant when selecting the surgical repair technique.

**Supplementary Information:**

The online version contains supplementary material available at https://doi.org/10.1007/s00423-026-04212-1.

## Introduction

Rotator cuff tears are among the most common causes of shoulder pain and dysfunction. Their prevalence in the general population may reach approximately 20%, imposing a substantial burden on public health systems and markedly impairing patients’ quality of life [1–3]. Such injuries are often accompanied by severe tendon degeneration, fatty infiltration, muscle atrophy, and pronounced tendon retraction. These pathological changes substantially increase the risk of retear after repair and represent major factors affecting long-term functional recovery and clinical outcomes [4]. With population ageing and increasing expectations regarding physical function and quality of life, the diagnosis and treatment of rotator cuff injuries have become increasingly important in sports medicine and orthopaedic practice. For patients with full-thickness rotator cuff tears or an inadequate response to conservative treatment, surgical intervention remains the standard approach for restoring shoulder biomechanics and function [5, 6]. In recent years, arthroscopic rotator cuff repair has gradually replaced traditional open surgery as the predominant surgical approach. Its primary objective is to achieve anatomical healing and mechanical stability at the tendon-bone interface, thereby reducing the risk of postoperative retear and providing durable and reliable functional improvement [7–9].

Among current rotator cuff repair techniques, single-row repair and double-row suture-bridge repair are two of the most widely used approaches. The single-row technique is relatively straightforward to perform, with tendon fixation achieved using a single row of anchors, and it can provide satisfactory early clinical outcomes. However, it also has important limitations, including a smaller tendon-bone contact area and greater stress concentration around the anchors. These factors may increase the risk of postoperative retear, particularly in patients with massive tears or osteoporosis [10, 11]. The double-row suture-bridge technique was developed to address these limitations. By using medial and lateral rows of anchors together with sutures spanning the footprint, this technique substantially increases tendon-bone contact area and promotes a more uniform distribution of pressure across the footprint [12, 13]. From a biomechanical perspective, double-row fixation provides greater initial fixation strength, broader footprint coverage, and reduced stress at the suture-bone interface, thereby creating a more favourable mechanical and biological environment for tendon-bone healing [14]. Although several meta-analyses and randomised controlled trials have shown that the DR technique generally provides superior structural healing compared with the SR technique, whether this anatomical advantage consistently translates into better functional outcomes remains uncertain. Moreover, the patient populations most likely to derive clinically meaningful benefits from DR repair have not been clearly established. Some studies have suggested that the functional advantages of DR repair may not reach the threshold for clinical significance in all patients, particularly those with small-to-medium tears. In addition, the potential benefits of DR repair must be weighed against its longer operative time and greater cost [15, 16].

Accordingly, the key knowledge gap addressed in this study is not whether DR repair is universally superior to SR repair, but rather whether the relative treatment effect differs according to preoperative tear size. We hypothesised that the clinical benefits of the DR technique would be most pronounced in patients with large-to-massive tears, whereas its advantages in patients with small-to-medium tears would be more limited. By providing more detailed subgroup-specific evidence, this retrospective study aims to support clinically informed selection of repair techniques according to individual tear characteristics.

## Methods

We retrospectively analyzed 150 patients with rotator cuff tears who were admitted to our department between January 2021 and September 2024. Patients were divided into the double-row (DR) suture-bridge group and the single-row (SR) suture-bridge group according to the surgical technique used. The inclusion criteria were as follows: (1) age > 18 years; (2) unilateral rotator cuff tear confirmed by magnetic resonance imaging (MRI); (3) failure of conservative treatment for at least 3 months; (4) ability to undergo and cooperate with arthroscopic surgery; and (5) voluntary participation with signed informed consent. The exclusion criteria were as follows: (1) irreparable rotator cuff tear; (2) previous surgery on the ipsilateral shoulder; (3) severe dysfunction of major organs, such as the heart, lungs, or brain, precluding surgical treatment; (4) systemic connective tissue diseases, such as rheumatoid arthritis; (5) severe osteoporosis; (6) requirement for tendon transplantation during surgery; (7) concomitant shoulder osteoarthritis, fracture, or infection; and (8) a follow-up period of < 12 months. This study was approved by the Ethics Committee of Hunan Medical University General Hospital (approval No. 20201005). The requirement for informed consent for participation in this retrospective study was waived by the Ethics Committee because all data were anonymized. The study was conducted in accordance with the Declaration of Helsinki.

### Preoperative management

Baseline characteristics, including age, sex, affected side, body mass index (BMI), and tear size, were systematically collected before surgery. All patients underwent standard radiographic and MRI examinations of the shoulder, and the severity of the rotator cuff tear was assessed based on the MRI findings. All procedures were performed by two orthopedic surgeons with more than 10 years of experience in shoulder arthroscopy to ensure consistency in surgical technique. To minimize assessment bias, postoperative pain and functional outcomes were independently evaluated by another senior orthopedic surgeon who was blinded to the treatment group.

### Surgical procedure

SR group: After successful induction of general anesthesia, the patient was positioned on the unaffected side, with the affected upper limb maintained at 45° of abduction and approximately 5 kg of traction applied to adequately expose the shoulder joint. The surgical field was routinely disinfected and draped. A standard posterior viewing portal was first established through the posterior “soft spot,” and a 30° arthroscope was introduced to inspect the glenohumeral joint. The extent of the rotator cuff tear, integrity of the long head of the biceps tendon, and condition of the glenoid labrum were comprehensively assessed. An anterior portal was subsequently established, and the arthroscope was advanced into the subacromial space. A radiofrequency ablation device and burr were used to clear the operative field and adequately expose the rotator cuff, subacromial surface, and footprint of the greater tuberosity of the humerus. The morphology and size of the tear, degree of tendon retraction, and tissue quality were then assessed under direct visualization. A radiofrequency ablation device was used to debride necrotic tissue at the tear margins and release adhesions to restore tendon mobility. A spherical burr was then used to decorticate the footprint of the greater tuberosity until a uniform bleeding bone bed was obtained. Depending on the tear size and bone quality, one to three anchors loaded with high-strength sutures were selected. Bone tunnels were prepared approximately 5 mm lateral to the footprint and adjacent to the articular cartilage margin, and the anchors were inserted at an angle of approximately 45°. Stable anchor fixation and smooth suture sliding were subsequently confirmed. Using a suture passer, the anchor sutures were passed through the tendon in a simple suture configuration, with the suture sites positioned approximately 1.0–1.5 cm from the tear margin to ensure adequate tissue purchase. Under arthroscopic visualization, the sutures were tied sequentially, with tension adjusted to achieve close apposition of the tendon to the prepared bone bed and restore anatomical coverage of the footprint. The excess sutures were then trimmed. After completion of the repair, the shoulder was passively moved through different angles to assess construct integrity and confirm the absence of gap formation or suture cut-through. The surgical field was thoroughly irrigated with normal saline, and meticulous hemostasis was achieved. The incisions were then closed in layers and covered with sterile dressings.

**Fig. 1:**
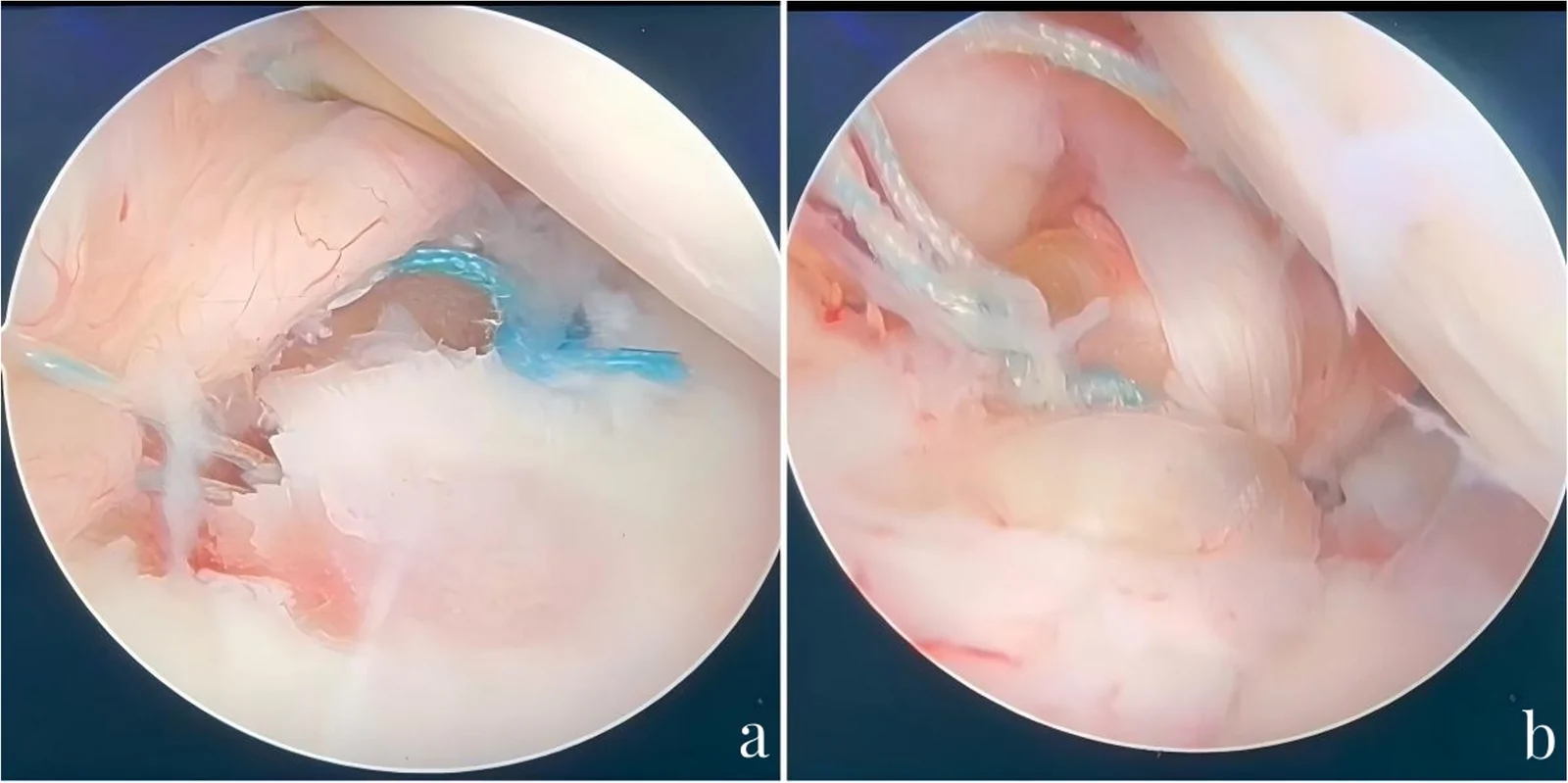
Arthroscopic suturing of the rotator cuff

**Fig. 2:**
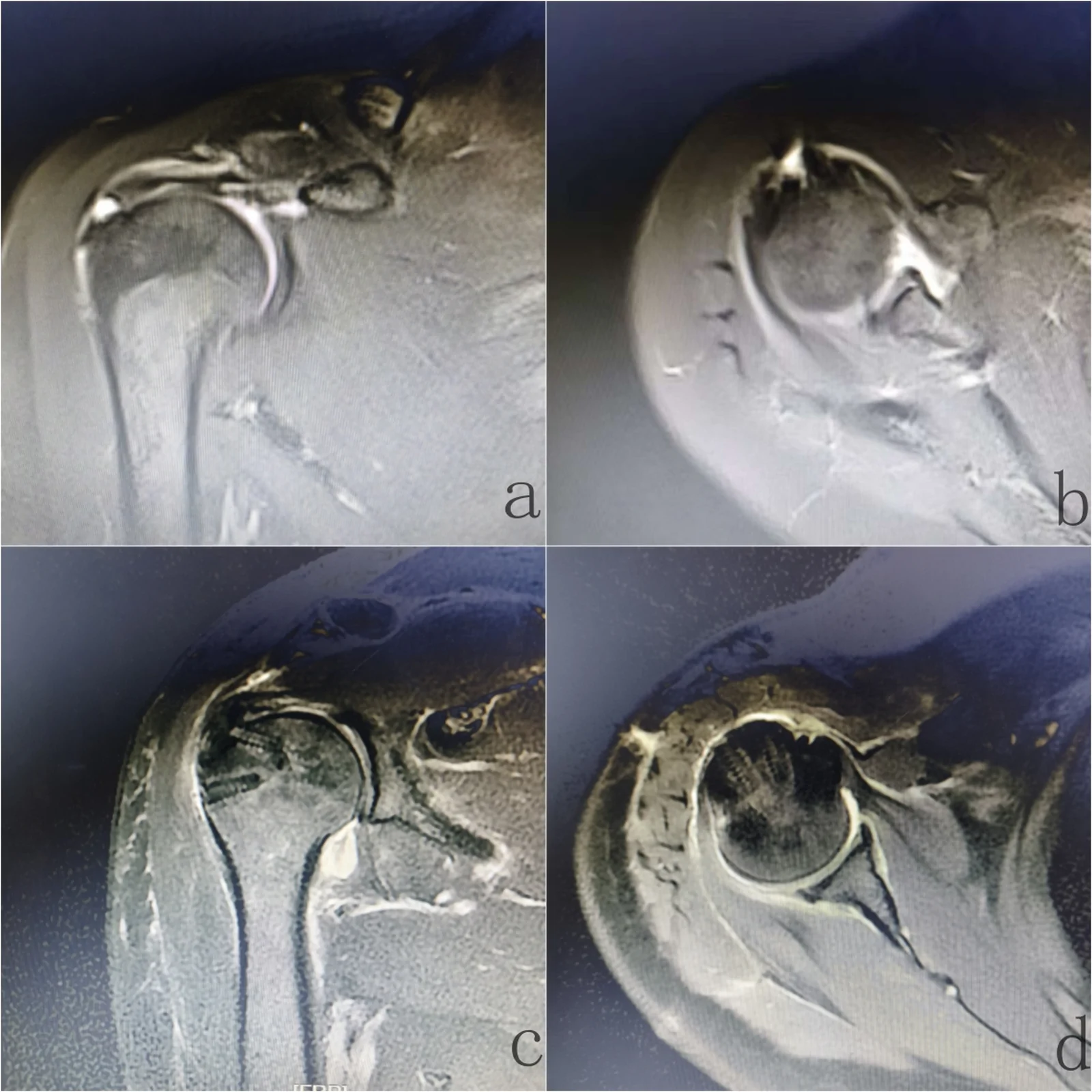
A 47-year-old male patient diagnosed with a rotator cuff tear and treated using the arthroscopic double-row suture-bridge technique. a, b: Preoperative MRI showing a rotator cuff tear and bone marrow edema of the greater tuberosity of the humerus. c, d: Follow-up imaging after double-row suture-bridge fixation showing good recovery

DR group: After completion of rotator cuff preparation, tendon release, and preparation of the footprint, a double-row reconstruction was performed. Two medial-row anchors, each loaded with two pairs of high-strength sutures, were inserted at the anterior and posterior aspects of the medial edge of the greater tuberosity footprint adjacent to the articular cartilage. The distance between the two anchors was maintained at approximately 1.5–2.0 cm. Each suture was then passed through the tendon in a horizontal mattress configuration using a suture passer, ensuring an even distribution of suture points. Under arthroscopic visualization, the medial-row sutures were sequentially tied to achieve initial close apposition of the torn tendon to the medial portion of the prepared bone bed. The suture limbs from the medial-row anchors were subsequently paired and crossed toward the lateral aspect of the greater tuberosity. Two knotless lateral-row anchors were inserted approximately 1 cm lateral to the footprint in alignment with the direction of the sutures. While maintaining appropriate tension between the rotator cuff tendon and the bone surface, the anchors were secured to create a stable suture-bridge construct over the tendon. This configuration provided uniform compression of the tendon across the entire prepared footprint (Fig. 1). After repair, the construct was inspected to confirm secure fixation and stability of the tendon-bone interface. The incisions were then routinely closed and covered with sterile dressings (Fig. 2).

Patients in both groups underwent the same standardized postoperative rehabilitation protocol. During the first 6 weeks after surgery, patients were instructed to wear a shoulder abduction brace for protection and to perform primarily passive rehabilitation exercises. These exercises included passive external rotation within the pain-tolerable range, with the angle gradually increased to 45°, as well as pendulum and circular exercises assisted by the unaffected upper limb. From 6 weeks to 3 months postoperatively, rehabilitation progressed to active-assisted and passive range-of-motion exercises. As tolerated, passive elevation, external rotation, and stretching exercises were gradually introduced. Beginning 3 months after surgery, patients initiated systematic strengthening exercises for the rotator cuff muscles and gradually resumed activities of daily living and light physical exercise.

## Outcome measures

Clinical outcomes were evaluated using the following measures: (1) Imaging-based healing and structural integrity: Magnetic resonance imaging (MRI) was performed 12 months postoperatively, and rotator cuff integrity was assessed according to the Sugaya classification [17]. Sugaya grades I–III were defined as structural healing, whereas grades IV and V were considered retears. The corresponding retear rates were calculated. (2) Shoulder function: The Constant-Murley score (CMS) [18], American Shoulder and Elbow Surgeons (ASES) score [19], and University of California at Los Angeles (UCLA) shoulder score [20] were used to systematically assess shoulder function preoperatively and 12 months postoperatively. (3) Pain intensity: The visual analogue scale (VAS) was used to assess pain at rest and during activity preoperatively and 12 months postoperatively. (4) Shoulder range of motion: Forward elevation, abduction, and external rotation of the shoulder were measured at the same time points using a long-arm goniometer. (5) Perioperative outcomes and safety: Operative time, intraoperative blood loss, length of hospital stay, and all postoperative complications were recorded and compared between the two groups.

### Subgroup stratification and additional statistical analysis

To investigate whether treatment effects differed according to tear size, patients were stratified into two subgroups based on preoperative MRI findings: the small-to-medium (SM) tear subgroup, comprising patients with small or medium tears, and the large-to-massive (LM) tear subgroup, comprising patients with large or massive tears.

To assess the clinical relevance of the observed differences, we calculated the proportion of patients in each group who achieved the minimal clinically important difference (MCID) for the ASES score. Based on previously published literature, an improvement of 21 points in the ASES score from baseline to the 12-month follow-up was defined as achievement of the MCID [21]. In addition, the number needed to treat (NNT) with the DR technique, compared with the SR technique, to prevent one retear was calculated for both the overall cohort and the LM subgroup.

### Statistical analysis

All statistical analyses were performed using SPSS version 25.0 (IBM SPSS Statistics, Chicago, IL, USA). The normality of continuous variables was assessed using the Shapiro-Wilk test. Normally distributed continuous variables are presented as the mean ± standard deviation. Between-group comparisons were performed using the independent-samples t-test, whereas within-group comparisons of preoperative and postoperative values were conducted using the paired t-test. Categorical variables are presented as frequencies and percentages and were compared using the χ² test. A P value < 0.05 was considered statistically significant.

## Results

A total of 150 patients were initially enrolled in the study. According to the prespecified exclusion criteria, two patients were excluded because of previous ipsilateral shoulder surgery, three because their follow-up duration was < 12 months, and one because tendon grafting was required intraoperatively. Consequently, 144 patients completed the study and were included in the final analysis. There were no statistically significant differences between the two groups in sex, age, affected side, tear size, BMI, or other key baseline characteristics (all *P* > 0.05). Detailed baseline characteristics are presented in Table 1. 

**Table 1:** Comparison of baseline data between the two groups of patients

	Control group (n=71)	Observation group (n=73)	Statistics	P value
**Gender (male/female)**	41/30	39/34	0.272	0.602
**Age**	47.72 ±7.76	48.37 ±7.48	0.502	0.616
**Injury side (left/right)**	28/43	33/40	0.491	0.484
**Tear size**			0.304	0.959
Small tear	23	21		
Medium tear	28	30		
Large tear	17	18		
Massive tear	3	4		
**BMI**	23.72 ±2.23	23.22 ± 2.14	1.370	0.173

Note:*BMI* Body mass index

Regarding perioperative outcomes, the operative time was longer in the DR group than in the SR group. Intraoperative blood loss was also slightly greater in the DR group. However, the absolute difference in blood loss between the groups was small and was unlikely to be clinically meaningful. No significant difference was observed in the length of hospital stay between the two groups. Detailed data are presented in Table 2.

**Table 2:** Comparison of perioperative indicators between the two groups of patients

	Control group(n=71)	Observation group(n=73)	Statistics	P value
Operation time (min)	60.41±5.10	72.95±4.78	15.226	<0.001
Blood loss (ml)	62.07±3.88	63.80±3.71	2.744	0.007
Hospital stay (day)	7.31±1.05	7.44±1.22	0.675	0.501

As shown in Table 3, all pain and functional scores improved significantly from baseline in both groups after surgery (*P* < 0.05). At the final follow-up, the DR group had significantly better VAS, CMS, UCLA, and ASES scores than the SR group, with all between-group differences reaching statistical significance (*P* < 0.001).

**Table 3:** Comparison of shoulder joint pain and functional scores between the two groups of patients

		Control group(n=71)	Observation group(n=73)	Statistics	P value
VAS	Preoperative	5.27±1.04	5.45±0.97	1.099	0.274
	Postoperation	1.89±0.77a	1.34±0.53a	4.966	<0.001
CMS	Preoperative	45.23±2.28	45.07±2.84	0.365	0.716
	Postoperation	77.79±2.90a	83.12±3.25a	10.381	<0.001
UCLA	Preoperative	11.08±1.32	11.15±1.56	0.275	0.784
	Postoperation	22.51±1.89a	25.85±2.34a	9.048	<0.001
ASES	Preoperative	41.79±2.32	42.11±1.98	0.895	0.372
	Postoperation	76.10±3.27a	82.05±2.91a	11.563	<0.001

Shoulder range of motion improved significantly from baseline in both groups after surgery (*P* < 0.05). At the final follow-up, forward elevation, abduction, and external rotation were significantly greater in the DR group than in the SR group (all *P* < 0.001), as shown in Table 4.

**Table 4:** Comparison of shoulder joint range of motion before and after surgery in the two groups of patients

		Control group(n=71)	Observation group(n=73)	Statistics	P value
Anterior elevation	Preoperative	81.35±2.51	80.78±2.45	1.384	0.168
	Postoperation	114.03±4.55b	119.27±3.38b	7.867	<0.001
Abduction angle	Preoperative	71.11±2.39	70.73±2.45	0.979	0.716
	Postoperation	81.66±1.86b	90.32±2.28b	24.892	<0.001
External rotation	Preoperative	27.23±2.11	27.86±2.27	1.742	0.084
	Postoperation	40.38±1.56b	42.99±2.11b	8.402	<0.001

As shown in Table 5, the overall retear rate was significantly lower in the DR group than in the SR group (4.11% vs. 16.90%; X^2^ = 6.311, *P* = 0.012). Notably, all retears observed in the DR group were classified as small or medium, whereas two large retears occurred in the SR group.

**Table 5:** Comparison of retear situations in the two groups of patients after surgery

	Small retear	Medium retear	Large retear	Total
Control group(n=71)	6(8.45%)	4(5.63%)	2(2.82%)	12(16.90%)
Observation group(n=73)	2(2.74%)	1(1.37%)	0(0%)	3(4.11%)
Statistics				6.311
P value				0.012

The superior outcomes observed in the DR group were not consistent across all tear sizes. As shown in Table 6, among patients with small-to-medium (SM) tears, functional outcomes at the final follow-up, including ASES, CMS, and UCLA scores, did not differ significantly between the SR and DR groups (all *P* > 0.05). In contrast, among patients with large-to-massive (LM) tears, the DR technique was associated with significantly better functional scores (all *P* < 0.01) and a markedly lower retear rate than the SR technique (0% vs. 30.0%, *P* = 0.007). For the ASES MCID, a significantly greater proportion of patients in the overall DR group achieved the clinically meaningful threshold than in the SR group (86.30% vs. 71.83%, *P* = 0.033). This difference was primarily attributable to the LM subgroup, in which 95.45% of patients treated with the DR technique achieved the MCID compared with 70.00% of those treated with the SR technique (*P* = 0.041). In contrast, no significant difference in MCID achievement was observed between the two techniques in patients with SM tears (Table 6). The NNT with the DR technique to prevent one retear was 7.8 for the overall cohort. More importantly, in the high-risk LM subgroup, the NNT was only 3.3, indicating a substantial preventive benefit of DR repair in patients with larger tears.

**Table 6:** Subgroup Analysis of Functional Outcomes and Retear Rates According to Tear Size

	Small-to-Medium Tears			Large-to-Massive Tears		
	SR Group (n=51)	DR Group (n=51)	P	SR Group (n=20)	DR Group (n=22)	P
ASES Score	78.52 ± 3.53	80.13 ± 3.83	0.147	69.2 2± 4.93	85.13 ± 4.16	<0.001
CMS	78.91 ± 2.82	80.06 ± 2.93	0.089	72.15 ± 4.53	83.93± 3.764	<0.001
UCLA	23.12 ± 1.73	24.02 ± 1.95	0.067	20.57 ± 2.12	26.92 ± 2.31	<0.001
Retear Rate	9 (17.65%)	3 (5.88%)	0.065	6 (30.00%)	0 (0%)	0.007
Achieved MCID for ASES	37 (72.55%)	42(82.35%)	0.236	14(70.00%)	21(95.45%)	0.041

Note: *CMS* Constant-Murley score, *UCLA* University of California at Los Angeles Shoulder score, *ASES* American Shoulder and Elbow Surgeons Score

## Discussion

Rotator cuff tears are a major cause of shoulder pain and dysfunction. Surgical treatment has evolved substantially from open repair to fully arthroscopic techniques. Throughout this evolution, achieving secure anatomical healing at the tendon-bone interface and reducing the risk of postoperative retear have remained central challenges in sports medicine and orthopaedic surgery [22, 23]. Repair strategies have progressed from single-row fixation to double-row techniques designed to increase footprint coverage, and subsequently to suture-bridge constructs that combine biomechanical advantages with practical feasibility. Each refinement in repair strategy has prompted renewed evaluation of its clinical effectiveness. The present study confirms the established biomechanical advantages of the double-row (DR) suture-bridge technique, demonstrating better overall functional outcomes and a lower retear rate than the single-row (SR) technique at 12 months of follow-up. Importantly, however, the principal contribution of this study lies not simply in confirming these overall advantages, but in demonstrating that the magnitude of benefit is strongly dependent on the initial tear size.

In the present study, ASES, Constant-Murley, and UCLA scores at the 12-month follow-up were significantly better in the DR group than in the SR group. This clinical advantage may be closely related to the biomechanical characteristics of double-row fixation. By creating a bridging construct between medial- and lateral-row anchors, the DR technique increases tendon-bone contact area and promotes a more uniform distribution of load across the repair site. This configuration may enhance the initial strength of the repair and reduce stress concentration at the suture-tendon interface [24, 25]. Previous biomechanical studies support this interpretation, showing that double-row repair provides greater footprint coverage than single-row repair and achieves higher failure loads during cyclic loading [26]. The DR group also demonstrated greater improvements in pain, as reflected by VAS scores, and in shoulder range of motion, including forward elevation, abduction, and external rotation. These improvements may result from more stable anatomical reconstruction, which may help restore the force-couple balance of the rotator cuff, reduce abnormal glenohumeral translation, and limit secondary inflammation. In this way, improved structural stability may provide a more favorable basis for functional recovery. Some previous studies have reported only modest differences in function between SR and DR repair during the early postoperative period, such as at 3 months, whereas the advantages of DR repair become more apparent at 6–12 months. This pattern suggests that DR fixation may provide greater benefits for maintaining structural integrity and supporting medium-term functional recovery [27].

This study also showed that the anatomical healing rate at 12 months was higher in the DR group, with a correspondingly lower retear rate than in the SR group. This finding is consistent with the original biomechanical rationale for double-row suture-bridge repair. By connecting the medial and lateral rows of anchors through bridging sutures, the technique provides broader compression of the rotator cuff tendon against the footprint, creating a larger tendon-bone contact surface than is generally achieved with single-row fixation. This increased contact area may provide a broader biological interface for cellular infiltration and collagen integration. In addition, the suture-bridge configuration may redistribute stress across the repair site, reducing localized stress concentrations that could otherwise contribute to suture cut-through or anchor loosening [28, 29]. This optimized mechanical environment may provide two potential advantages. First, greater initial fixation strength may allow the repair construct to better withstand repetitive loading during early rehabilitation, thereby protecting the tendon-bone interface during the initial healing period. Second, broader compression and more uniform load distribution may create a more favorable healing environment, particularly in technically demanding tears. Our findings are consistent with those of several recent meta-analyses reporting improved structural integrity with double-row or suture-bridge repair [30, 31]. Nevertheless, these proposed mechanisms remain biologically and biomechanically plausible interpretations rather than direct mechanistic findings from the present clinical study and therefore require further validation.

The subgroup analysis provides the most clinically relevant finding of this study. The advantages of the DR technique in both functional recovery and structural healing were concentrated predominantly in patients with large-to-massive (LM) tears. In this high-risk subgroup, DR repair reduced the retear rate from 30% to 0% and produced improvements in functional outcomes that were both statistically and clinically meaningful, as reflected by the higher proportion of patients achieving the minimal clinically important difference (MCID). In contrast, among patients with small-to-medium (SM) tears, DR repair did not demonstrate a clear clinically meaningful advantage over SR repair. Given that SR repair is technically simpler, requires less operative complexity, and is likely to be less costly, these findings do not support the routine use of DR repair for all tear sizes. Instead, they favor a more selective, risk-stratified approach to surgical technique.

Our findings are consistent with the recent meta-analysis by Perez Gutierrez et al. [32], while extending the available evidence by providing tear-size-specific subgroup data that may be more directly applicable to clinical decision-making. Previous meta-analyses have generally demonstrated an overall structural advantage of DR repair, but they have provided limited guidance regarding which individual patients are most likely to derive a clinically meaningful benefit. By estimating the number needed to treat (NNT) for prevention of one retear, our study provides a more clinically interpretable measure of treatment effect. The NNT was 3.3 in patients with LM tears, indicating a substantial potential benefit in this high-risk subgroup, whereas the corresponding advantage was considerably less apparent in patients with SM tears. These findings suggest that the additional footprint restoration and mechanical stability provided by DR repair may be most important when the demands on the repair construct are greatest, as in larger tears with broader defects and a less favorable healing environment.

Several limitations should be considered when interpreting these findings. First, the retrospective and non-randomized design introduces the possibility of selection bias. Although baseline characteristics were comparable between the two groups, the choice of surgical technique was not randomized, and unmeasured confounding factors, including surgeon preference, intraoperative findings, and subtle differences in tear morphology, may have influenced the results. Second, this was a single-center study with a relatively modest sample size of 144 patients. This limits the statistical power of the subgroup analyses, particularly for massive tears, and may restrict the generalizability of the findings to other populations and clinical settings. Third, the 12-month follow-up period, although sufficient to assess early tendon healing and functional recovery, does not allow evaluation of longer-term outcomes such as delayed retear, progression of degenerative changes, or the durability of functional improvement. Fourth, bone quality and tear chronicity were not systematically quantified, despite their known influence on anchor fixation and tendon healing. Fifth, retear was assessed using MRI at a single postoperative time point, and the clinical significance of structural failure, particularly small retears, remains uncertain. Finally, although postoperative functional assessments were performed by a blinded evaluator, blinding of patients and surgeons was not feasible because of the nature of the surgical intervention, potentially introducing performance bias. These findings should therefore be validated in larger, prospective, multicenter randomized controlled trials with longer follow-up.

## Conclusion

In conclusion, arthroscopic double-row suture-bridge repair was associated with better overall structural healing and functional recovery than single-row repair. However, subgroup analysis showed that this advantage was driven predominantly by patients with large-to-massive rotator cuff tears. In patients with small-to-medium tears, the DR technique did not demonstrate a clear clinically meaningful benefit over SR repair. These findings support a more individualized surgical strategy in which tear size is considered an important determinant of repair technique, with the more complex DR approach preferentially considered for patients at greater risk of retear.

## Supplementary Information

Below is the link to the electronic supplementary material.

Supplementary Material 1

## Data Availability

No datasets were generated or analysed during the current study.

## References

[1] Bedi A, Bishop J, Keener J et al (2024) Rotator cuff tears. Nat Rev Dis Primers 10(1):8 10.1038/s41572-024-00492-3 38332156

[2] Alben MG, Gambhir N, Papalia AG et al (2023) Return to sport after arthroscopic rotator cuff repair of full-thickness rotator cuff tears: A systematic review. Clin J Sport Med 33(4):440–449 10.1097/JSM.0000000000001130 36853904

[3] Yamamoto A, Takagishi K, Osawa T et al (2010) Prevalence and risk factors of a rotator cuff tear in the general population. J Shoulder Elb Surg 19(1):116–120 10.1016/j.jse.2009.04.006 19540777

[4] Oh JH, Song BW, Lee YS (2014) Measurement of volumetric bone mineral density in proximal humerus using quantitative computed tomography in patients with unilateral rotator cuff tear[J]. J Shoulder Elb Surg 23(7):993–1002 10.1016/j.jse.2013.09.024 24388151

[5] Longo UG, Risi Ambrogioni L, Candela V et al (2021) Conservative versus surgical management for patients with rotator cuff tears: a systematic review and META-analysis. BMC Musculoskelet Disord 22(1):50 10.1186/s12891-020-03872-4 PMC7796609 33419401

[6] Lapner P, Henry P, Athwal GS et al (2022) Treatment of rotator cuff tears: a systematic review and meta-analysis. J Shoulder Elb Surg 31(3):e120–e129 10.1016/j.jse.2021.11.002 34906681

[7] Plachel F, Jo OI, Rüttershoff K et al (2023) A systematic review of long-term clinical and radiological outcomes of arthroscopic and open/mini-open rotator cuff repairs. Am J Sports Med 51(7):1904–1913 10.1177/03635465211073332 35179393

[8] Zhao J, Luo M, Pan J et al (2021) Risk factors affecting rotator cuff retear after arthroscopic repair: a meta-analysis and systematic review. J Shoulder Elb Surg 30(11):2660–2670 10.1016/j.jse.2021.05.010 34089878

[9] Henry P, Wasserstein D, Park S et al (2015) Arthroscopic repair for chronic massive rotator cuff tears: A systematic review. Arthroscopy 31(12):2472–2480 10.1016/j.arthro.2015.06.038 26364549

[10] Greenspoon JA, Petri M, Warth RJ et al (2015) Massive rotator cuff tears: pathomechanics, current treatment options, and clinical outcomes. J Shoulder Elb Surg 24(9):1493–1505 10.1016/j.jse.2015.04.005 26129871

[11] Gartsman GM, Drake G, Edwards TB et al (2013) Ultrasound evaluation of arthroscopic full-thickness supraspinatus rotator cuff repair: single-row versus double-row suture bridge (transosseous equivalent) fixation. Results of a prospective, randomized study. J Shoulder Elb Surg 22(11):1480–1487 10.1016/j.jse.2013.06.020 24012360

[12] Huang AL, Thavorn K, van Katwyk S et al (2017) Double-row arthroscopic rotator cuff repair is more cost-effective than single-row repair. J Bone Joint Surg Am 99(20):1730–1736 10.2106/JBJS.16.01044 29040127

[13] Kim SJ, Kim SH, Moon HS et al (2016) Footprint contact area and interface pressure comparison between the knotless and knot-tying Transosseous-equivalent technique for rotator cuff repair. Arthroscopy 32(1):7–12 10.1016/j.arthro.2015.07.004 26382638

[14] Franceschi F, Papalia R, Franceschetti E et al (2016) Double-row repair lowers the retear risk after accelerated rehabilitation. Am J Sports Med 44(4):948–956 10.1177/0363546515623031 26797698

[15] Bedeir YH, Schumaier AP, Abu-Sheasha G et al (2019) Type 2 retear after arthroscopic single-row, double-row and suture bridge rotator cuff repair: a systematic review. Eur J Orthop Surg Traumatol 29(2):373–382 10.1007/s00590-018-2306-8 30229445

[16] Lapner P, Li A, Pollock JW et al (2021) A multicenter randomized controlled trial comparing single-row with double-row fixation in arthroscopic rotator cuff repair: Long-term follow-up. Am J Sports Med 49(11):3021–3029 10.1177/03635465211029029 PMC8411465 34398641

[17] Sugaya H, Maeda K, Matsuki K et al (2005) Functional and structural outcome after arthroscopic full-thickness rotator cuff repair: single-row versus dual-row fixation[J]. Arthroscopy 21(11):1307–1316 10.1016/j.arthro.2005.08.011 16325080

[18] Collin P, Betz M, Herve A et al (2020) Clinical and structural outcome 20 years after repair of massive rotator cuff tears. J Shoulder Elb Surg 29(3):521–526 10.1016/j.jse.2019.07.031 31594728

[19] Agel J, Hebert-Davies J, Braman JP (2023) American shoulder and elbow surgeons score: What does it tell us about patients selecting operative treatment of a rotator cuff injury[J]? JSES Int 7(5):751–755 10.1016/j.jseint.2023.04.007 PMC10499637 37719827

[20] Elliott RSJ, Lim YJ, Coghlan J et al (2019) Structural integrity of rotator cuff at 16 years following repair: Good long-term outcomes despite recurrent tears. Shoulder Elb 11(1):26–34 10.1177/1758573217738198 PMC6348585 30719095

[21] Lee AC, Chiang J, Li X et al (2026) A novel methodology for establishing best values for MCID, SCB, and PASS thresholds for rotator cuff repair. Am J Sports Med 54(3):748–757 10.1177/03635465251380288 41502123

[22] Chalmers PN, Granger E, Nelson R et al (2018) Factors affecting cost, outcomes, and tendon healing after arthroscopic rotator cuff repair. Arthroscopy 34(5):1393–1400 10.1016/j.arthro.2017.11.015 29371013

[23] Goetti P, Denard PJ, Collin P et al (2020) Shoulder biomechanics in normal and selected pathological conditions. EFORT Open Rev 5(8):508–518 10.1302/2058-5241.5.200006 PMC7484714 32953136

[24] Lin Y, Zhao J, Qiu H et al (2020) Comparison of arthroscopic single-row and double-row repair in the treatment of rotator cuff tears: A protocol of cohort analysis. Med (Baltim) 99(29):e21030 10.1097/MD.0000000000021030 PMC7373522 32702843

[25] Hantes ME, Ono Y, Raoulis VA et al (2018) Arthroscopic single-row versus double-row suture bridge technique for rotator cuff tears in patients younger than 55 Years: A prospective comparative study. Am J Sports Med 46(1):116–121 10.1177/0363546517728718 28942685

[26] He HB, Hu Y, Li C et al (2020) Biomechanical comparison between single-row with triple-loaded suture anchor and suture-bridge double-row rotator cuff repair. BMC Musculoskelet Disord 21(1):629 10.1186/s12891-020-03654-y PMC7517672 32972397

[27] Urch E, Lin CC, Itami Y et al (2020) Improved rotator cuff footprint contact characteristics with an augmented repair construct using lateral edge fixation. Am J Sports Med 48(2):444–449 10.1177/0363546519888182 31800297

[28] Gu Z, Wu S, Yang Y et al (2023) Comparison of arthroscopic single-row and double-row repair for rotator cuff injuries with different tear sizes: A systematic review and meta-analysis. Orthop J Sports Med 11(8):23259671231180854 10.1177/23259671231180854 PMC10467404 37655249

[29] Sheean AJ, Hartzler RU, Burkhart SS (2018) Rotator cuff repair: Single row repair versus double row repair and superior capsular reconstruction. Sports Med Arthrosc Rev 26(4):171–175 10.1097/JSA.0000000000000228 30395062

[30] Hu CW, Tsai SHL, Chen CH et al (2023) Early versus delayed mobilization for arthroscopic rotator cuff repair (small to large sized tear): a meta-analysis of randomized controlled trials. BMC Musculoskelet Disord 24(1):938 10.1186/s12891-023-07075-5 PMC10694899 38049792

[31] Park MC, ElAttrache NS, Tibone JE et al (2007) Part I: Footprint contact characteristics for a transosseous-equivalent rotator cuff repair technique compared with a double-row repair technique. J Shoulder Elb Surg 16(4):461–468 10.1016/j.jse.2006.09.010 17321161

[32] Perez Gutierrez EA, Mendoza AA, Galaviz Morales JP et al (2025) Comparative analysis of single-row vs. double-row technique for rotator cuff repair: a systematic review and statistical analysis. JSES Rev Rep Tech 5(4):857–864 10.1016/j.xrrt.2025.05.017 PMC12573510 41179468

