# Supplementary material for: A retrospective study of the clinical efficacy of arthroscopic double-row and single-row suture-bridge techniques for rotator cuff tears
Source: Langenbecks Arch Surg. 2026 Aug 19;411(1):318. doi: 10.1007/s00423-026-04212-1 (PMC13768361; doi:10.1007/s00423-026-04212-1)
Supplement: Supplementary file 1 [file 423_2026_4212_MOESM1_ESM.pdf]

Certificate of English Editing

E-TRANSTAR (Beijing) Information Technology Co., Ltd.

Title

A retrospective study on the clinical efficacy of arthroscopic double-row and single-row suture bridge techniques in the treatment of rotator cuff tears

Authors

Bingqiang Shi, MDa, Bingling Shi, MDb, Yong Xu, MD c,§

Date of Editing

August 11, 2026

This document certifies that the paper listed above has been edited to ensure that the language is clear and free from errors. The edit was performed by

Dorothy

native English speaker editor of

E-transtar (Beijing) Information Technology Co., Ltd.

The intent of the author’s message was not altered in any way during the editing process. The quality of the edit has been guaranteed, with the assumption that our recommended changes have been accepted and have not been altered without the knowledge of our editors.

Certificate issued by:
E-transtar (Beijing) Information Technology Co., Ltd.
